# Edge-enabled Mobile Crowdsensing to Support Effective Rewarding for Data Collection in Pandemic Events

**DOI:** 10.1007/s10723-021-09569-9

**Published:** 2021-07-08

**Authors:** Luca Foschini, Giuseppe Martuscelli, Rebecca Montanari, Michele Solimando

**Affiliations:** Department of Computer Science and Engineering (DISI), University of Bologna, Viale Risorgimento 2, 40136 Bologna, Italy

**Keywords:** Edge computing, Mobile crowd sensing, Smart city, Blockchain, Pandemic prevention

## Abstract

Smart cities use Information and Communication Technologies (ICT) to enrich existing public services and to improve citizens’ quality of life. In this scenario, Mobile CrowdSensing (MCS) has become, in the last few years, one of the most prominent paradigms for urban sensing. MCS allow people roaming around with their smart devices to collectively sense, gather, and share data, thus leveraging the possibility to capture the pulse of the city. That can be very helpful in emergency scenarios, such as the COVID-19 pandemic, that require to track the movement of a high number of people to avoid risky situations, such as the formation of crowds. In fact, using mobility traces gathered via MCS, it is possible to detect crowded places and suggest people safer routes/places. In this work, we propose an edge-anabled mobile crowdsensing platform, called ParticipAct, that exploits edge nodes to compute possible dangerous crowd situations and a federated blockchain network to store reward states. Edge nodes are aware of all critical situation in their range and can warn the smartphone client with a smart push notification service that avoids firing too many messages by adapting the warning frequency according to the transport and the specific subarea in which clients are located.

L. Foschini
e-mail: luca.foschini@unibo.it

G. Martuscelli
e-mail: giuseppe.martuscelli@unibo.it

R. Montanari
e-mail: rebecca.montanari@unibo.it

## 1 Introduction

Smart cities put digital technology at the service of citizens to improve their quality of life and of city rulers to better govern their service provisioning decisions and to improve sustainability. Smart city enabling technologies, such as Internet of Things (IoT) or cloud computing, promote innovation of city services in several fields, from urban transport, water supply, waste disposal facilities to more efficient building lightening and heating systems. Mobile CrowdSensing (MCS) represents currently another crucial technological enabler allowing the collection and sharing of great amounts of data and the monitoring/detection of citizenship habits and movements in urban environments. All sensors and human behavior collected data can be processed using machine learning algorithms and give us back higher-level inferences and useful information [1–3]. Especially in the recent COVID-19 pandemia, by enabling to enrich gathered data with location- and context-aware information, MCS can be helpful to support user's contact tracing and people's crowding degree computation in urban areas, a crucial information to limit the virus spread.

As key feature, with MCS data collection can be enabled from smartphones or tablets without the need to rely on a priori deployment of a network of traditional physical sensors, thus tearing down the cost and the time related to the design and construction of a sensor network. Two different approaches can be exploited to generate data. On the one hand, raw data can be gathered directly from the embedded sensors (GPS, camera, microphone, etc.) without user involvement, on the other hand, the user itself injects data of interest. The latter case considers the user as a sensor and is commonly known as social sensing to be used beside or alternatively to the physical sensors to allow users to enrich injected data with relevant details.

In any MCS system, the involvement of as many people as possible is crucial for the sensing campaign's success; a greater amount of information leads to more complete inferences, therefore to high-quality data sets. A largely used gimmick to increase and incentivize participation is through reward programs that loyalize and involve the users. The crowdsensing campaign can be proposed in a form of a game that favors the involvement of a great number of people, augmenting the quantity and the quality of the gathered information. In this way, the user is encouraged and stimulated to complete the task. The participants, accomplishing the sensing campaign's objectives, gain a price as a reward for their actions, which can be virtual, such as virtual points, or can be real such as a little amount of money useful to the users.

However, the management of large amount of data collected in smart city, especially in pandemia scenarios, as well as the effective support of rewarding become difficult to address with traditional cloud-based centralized MCS platforms requiring a shift toward novel architectures capable of moving computation where the end-user devices are more nearby. In particular, for massive scale MCS deployments Multi-access Edge Computing (MEC) is a promising recent architectural model and specification (i.e., by European Telecommunications Standards Institute - ETSI) that allows to add to the traditional two-layers MCS platform deployment model a third layer, at the edge of the network [4]. MEC adds resources and information at the peripheral of the network enabling the processing, filtering, and aggregation of real-time data close to data sources and allowing to reduce the latency in communication [5]. MEC servers can facilitate the control of the sensing process on mobile devices located within their deployment area and participate in the management of MCS tasks within the same area. The edge layer can leverage on their own resources to lighten the workload of both mobile devices and servers in cloud. Furthermore, the execution offloading on MEC nodes potentially reduces the complexity of any software platform's components running in the other architectural layers.

Taking into account the great potential of the joint exploitation of MEC and MCS solutions, this paper proposes and describes the adoption of MEC for MCS along two different directions. On the one hand, we propose a MEC-based MCS architecture for pandemic scenarios, such as the COVID-19 one, capable of leveraging the collection of data from mobile users and their analysis in order to identify the crowding degree of urban areas. Users who pass through the areas covered by the MEC nodes can benefit from timely notifications on the level of crowding in the nearby areas. In particular, the system performance is not affected by this additional calculation.The supplementary edge layer is responsible of finding cluster of people overcrowding the same area, thus relieving the server from the processing of great amount of geographical data. In addition, given the knowledge of data location and context, the use of MEC nodes improves the accuracy of the information notified to users. On the other hand, to improve the effectiveness of user's rewards we propose an edge-enabled distributed ledger architecture to record the reward assignments among untrusted and unknown participants in a generic gamification system. Leaning on ETSI MEC nodes, the platform exhibits high scalability because of the great availability of additional computational and storage resources on the edge used to execute distributed ledger-related functions. An edge layer between the participants and the cloud server could perform all needed tasks required to use a blockchain, leaving the cloud servers free to focus on their main crowdsensing core business processing. Moving the rewards on the edge nodes also gives redundancy and fault tolerance to the whole platform, avoiding the loss of all users' achievements due to a potential server critical fault. The adoption of an edge enabled blockchain also leverages the security of the whole system. Distributing the rewards through the blockchain on edge nodes prevents the stealing or faking of users' accomplishments resulting from an internal or external attack to the server itself. In particular, this twofold contribution of the paper has been designed, developed, and tested by extending our MCS framework called ParticipAct [6].

The paper is structured as follows. In Section 2 we present the state of art and the background related to Edge Computing in correlation with MCS and with blockchain to support emergency scenario, such as the COVID-19 pandemic; in Section 3 we present the edge-enabled parts of ParticipAct which, supported by the Edge, collects location information and after the aggregation process notifies potentially dangerous crowded areas in the proximity. Section 3 details also the edge-based distributed ledger architecture we have developed for supporting decentralized incentives in ParticipAct, while Section 4 focuses on our solution implementation and experimental results. Finally, Section 5 concludes the paper.

## 2 State of the Art and Background

This section is intended to provide a background overall view on the topics covered in this paper. In particular, we will present notable works found in the literature related to the edge support to mobile crowdsensing platforms and to distributed ledger deployments.

### 2.1 Mobile CrowdSensing and Multi-access Edge Computing

MCS has gained significant attention in recent years and has become an appealing paradigm for urban sensing. Thanks to the MCS paradigm, receiving heterogeneous contributions from the crowd of people becomes possible. Data collection is performed directly on devices owned by participants to the crowdsensing campaign. These devices range from dumb wearables terminals to more powerful smartphones and tablets. Although the computational power increases, the mobile devices used for MCS campaigns remain constrained in terms of autonomy and power supply. To involve a growing number of people, some form of rewards should be assured to the users for their good quality contributions. In this way, it is possible to increase the catchment area of the MCS platforms. The recent advancement in network architecture, with the addition of edge layer and edge computing capabilities, seems to facilitate the management of MCS complex platforms, opening different solutions that take care of locality and efficiency in executing and maintaining crowdsensing campaigns. In particular, the Multi-access Edge Computing [7], whose definition was specified by European Telecommunications Standards Institute (ETSI) is a natural choice for leveraging MCS platforms [13]. MCS platforms can exploit the most important features of MEC schema such as the computational power at the edge of the network (ideally one-hop from the end-user devices), achieving a very fast communication between services and participants, and breaking down the latencies that usually affect the cloud deployments. Ultra-low latency and large bandwidth result in more secure and reliable services, enriched with context-awareness and locality information [4]. The ETSI MEC reference architecture specifies all the components in the virtualized environment to provide developers with a complete IT service environment to run MEC applications on operator network [9]. We refer to a MEC node as the asset, at the edge of the network, having all the resources to execute applications, such as storage, processing, and networking. In the following, we present some works, found in the literature, that use the edge computing paradigm to aid crowdsensing operations and to ease the gathering of contributions and the distribution of rewards.

In [10] authors analyze the merge between MCS technologies and Mobile Edge Computing, the original definition of edge computing by ETSI, now replaced with Multi-access Edge Computing. The authors propose a new scalable architecture that relies on the edge layer for heavy computation and that pays much attention to the privacy of participants' data. The authors provide a use case scenario in which a crowdsensing application is used to enable neighborhood collaboration to improve the quality of life in urban districts. For example, if a user wants to know the traffic situation or she expresses the will of having a park area without pollution, she creates/joins a task for the neighborhood, giving other people the possibility to share their experiences and contributions. The work outlines several advantages of using mobile edge computing as an intermediate layer between clients and cloud servers. MEC contributes to reducing data processing and services execution, usually in charge of the server. Only aggregated and filtered (not redundant) data comes to the cloud. Many open issues need still to be solved to complete the full integration between MEC and MCS. The interoperability among different MCS platforms is assured only if they use the same interfaces, open communication protocols, and standard data models. Furthermore, giving user data to the edge layer opens the privacy issue about customers' profiles. Also, the cost of orchestrating the various MCS services among the edge nodes is not negligible.

In [11] an MCS agent-based service provided by smart objects is proposed that relies on agents on the edge of the network and on end user's devices. The work tackles the big problem of opportunistic resource provisioning, due to the unpredicted mobility of the users in such a scenario. Their solution, namely (Agent-oriented Cooperative Smart Object-Methodology) ACOSO-Meth, is a guideline that drives the developer in the implementation of a crowdsensing service starting from a systematical analysis of its own processes. The edge nodes executing the agents enable to perform context-aware operations of the crowdsensing platform. Authors state that the edge layer helps the system to save resources and covers the case of user mobility, giving dynamicity to the whole platform. In the work described in [11] standard Web interfaces and REST API calls are used to overcome the interoperability issue. The work shows the analysis, design, and implementation phases using the user mobility as a crowdsensing campaign use case. The IoT agents on the edge expose the API that agents on the smartphones can call to update or to know the status of the node.

In [12] the edge computing paradigm is introduced to process raw data, to improve the latency and to protect users' privacy. This need is especially dictated in the case of massive participation in crowdsensing campaigns. When the data to sense increases, the ability of the platform is limited and the privacy of crowds could be revealed. The authors break the classic two-layer deployment of a crowdsensing platform adding the edge layer to receive contextual users' contributions, for example, the user's position in a certain area. The edge servers take charge of computation-intensive tasks and represent the communication interface between participants and the cloud MCS platform. The edge nodes also filter raw information before sending them to the cloud server, deleting the noisy data and avoiding external network congestion and latencies. The distributed nature of edge computing allows the local storage of user-profiles, thus preserving their privacy and avoiding the transmission of sensitive information over the public Internet. Once the cloud server gives the task to the edge nodes, the reward for accomplishing a campaign is estimated by the edge and the users. The authors here elaborate on an efficient incentive mechanism designing a three-layer game for the platform. To decide who must perform the task, edge nodes play a game in which they have to maximize the gain. The task cost for each edge server is the payoff to the crowd in case of execution of the considered task. The reward to the users undergoes an adjustment, since the participants are moving, they can change spot while performing a task, so the authors considered a probability of termination when calculating the task cost for each edge node. In the end, the cloud server decides which node to assign the task based on the winner of the game among the edge nodes. The work ends by saying that this fair assignment mode is achievable thanks to edge computing, which performs many fast operations that the cloud server cannot cope with due to the higher latency.

## 2.2 Distributed Ledgers and Edge Computing

Blockchain acts as a distributed, decentralized and immutable append-only ledger that stores blocks of data containing transactions between nodes in a peer-to-peer (P2P) network. Blockchain technology provides a decentralised trust model in an environment where participants typically do not trust each other allowing the implementation of a tamper-proof ledger with characteristics of immutability, censorship resistance and transaction timestamping. There are currently several blockchain platforms that can be classified depending on different criteria [8]. For instance, based on access regulation blockchain can be divided into public and private. The public blockchains is an open network and anyone can join it without any approval and can publish and validate transactions. In private blockchain the participation is regulated by an owner who decides who can access the network. Blockchain networks can be also classified on the basis of the access models. In permissionless blockchain any peer can take part in the network and to be involved in the consensus process. In the other model, participation is limited and can be confined only on writing (validation) or reading rights. Among the most widespread permissionless blockchains we can find Bitcoin and Ethereum. Bitcoin is an open-source blockchain invented in 2008, it offers open access to the transactions and it is based on Proof-of-Work consensus algorithm, Ethereum is a decentralized open platform which supports natively smart contracts applications. This programs can execute automatically tasks such as money exchange when certain conditions are met. Solutions that belong to the permissioned blockchain category, instead, are Hyperledger Fabric project which is mostly supported by IBM and allows pluggable consensus protocols and Sawtooth which is contributed by Intel and introduce a Proof of Elapsed Time (PoET) consensus to consume less energy increasing efficiency.

The integration of blockchain and edge computing can take advantages from each other exploiting the security and privacy features offered by the blockchain and the possibility of scaling a distributed system offered by the edge computing paradigm [14]. In this way the integrated frameworks and functionalities of blockchain and edge computing-based systems can enable reliable access and control of the network, storage, and computation distributed at the edges, hence providing a large scale of network servers, data storage, and validity computation near the end in a secure manner. In particular, the incorporation of edge computing into blockchain brings the powerful decentralized network and rich computation and storage resources in the network edge. Conversely, the incorporation of blockchain into edge computing enhances the security, privacy, and the automatic resource usage. Using the blockchain technique, it is possible to build a distributed control at dozens of edge nodes. Thanks to the mining process and the replication on many nodes, blockchains protect the accuracy, consistency and validity of the data and rules over their life cycle in a transparent way. Despite the prospected benefits of integrated blockchain and edge computing systems, several issues remain to be addressed before widespread deployment.

Some studies are emerging that propose frameworks to integrate blockchain and edge computing systems with the most disparate goals and within dfferent application scenarios. Sharma et al. [15], for example, proposes a blockchain-based distributed cloud architecture with a software defined networking (SDN) enable controller fog nodes at the edge of the network to provide low-cost, secure, and on-demand access to the most competitive computing infrastructures in an IoT network.

Guo et al. [16] proposes a hybrid architecture to facilitate access control of Electronic Health Record (EHR) data by using both blockchain and edge node. Within the architecture, a blockchain-based controller manages identity and access control policies and serves as a tamper-proof log of access events. In addition, off-chain edge nodes store the EHR data and apply policies specified.

In [17], a novel blockchain-based security architecture in NDN Vehicular Edge Computing networks is introduced to systematically tackle their security, such as key management, cache poisoning, access control. More specifically, authors design and implement an efficient blockchain system on NDN by adopting lightweight yet robust delegate consensus algorithm and carry out extensive experiments to evaluate performance efficiency on key management protocols, cache poisoning defense schemes, and access control strategies for NDN-based VEC networks.

The solution described in [18] proposes a secure and efficient V2G energy trading framework by exploring the joint adoption of blockchain and edge computing. In particular, a consortium blockchain-based secure energy trading mechanism for V2G is developed and the edge computing has been incorporated to improve the successful probability of block creation.

In [19] the applicability of the integration of blockchain and edge has been described by considering different scenarios ranging from smart cities, smart transportation to Industrial IoTs, smart homes and sart grids.

### 2.3 MCS and Blockchain for Pandemia Management

The sensed data can converge in databases belonging to several domains, which can be neatly categorized as suggested in [20]. Crowd behavior, environment, and infrastructure monitoring and control are just some of the potential opportunities that MCS could open in favor of smart city services. The exploitation of MCS solutions in the environmental field aims to monitor environmental data, such as weather, air, noise pollution levels with the ultimate goal of basically preserving the nature. Infrastructure monitoring represents another prominent field for MCS platforms: large-scale sensing among citizens enable the prompt and fast identification of city outages, such as faults in the lighting system or in the city water supply and can also prevent traffic congestion and suggest parking areas. The benefits people gains from MCS solutions may also involve opinions about places and recommendations based on everyone's shared experience. Furthermore, MCS can be exploited to infer collective behavior patterns and to conclude about community intelligence. Within these domains, many solutions have been proposed and discussed. Much less research and real-case works have emerged in relation to the capability of MCS platforms to enhance emergency situation management and especially pandemia scenarios, being these cases of recent occurrences and extremely complex to address. Along this direction, the few existing works provide some insights by sharing the common idea to employ citizens in crowdsensing campaigns to keep under control the diffusion of infective diseases. In the work described in [21] authors present the timeline evolution of the COVID-19 pandemic in Spain, and summarise the MCS research efforts that are being undertaken by the Spanish community to address COVID-19 outbreak. In this study, some new developments within the MCS framework have been introduced to achieve the smart quarantine concept in Spain. Since the sensitivity of shared data, such as posts on social networks and GPS locations, the authors try to find a trade-off between the privacy of participants and the profit that the society derives from the accuracy and number of data collected. The authors of [22] propose a study for the acceptance of crowdsensing campaigns aimed at tracking infected people. The participatory and opportunistic capabilities of this idea allow to the creation of a city map about the places visited by infected citizens in order to identify location that need a more accurate disinfection, such as metro stops, squares, commercial business, offices. The study focuses on the willingness to share location information and health status related to the COVID-19 disease, through the exploiting of the aforementioned crowdsensing technique.

Similar considerations apply to the applicability of blockchain in the particular field of pandemia management. Whereas a great number of use-cases of blockchain adoption exist in various application domains, the benefit of blockchain for pandemia management have been only recently identified but experimentation is still lacking. We can exploit Blockchain mechanisms to tackle several use cases occurring during the current COVID-19 pandemic situation. For example, the clinical validation of vaccines and drugs can be a real application that would last even after the current health crisis. Furthermore, since its privacy-preserving features, the health authorities can use the Blockchain to transparently track blood or body organ donors and fundraising activities [23, 24]. Blockchain can also help to better manage supply chains that the COVID-19 crisis has rattled by helping rebuilding disrupted networks, by providing trading partners and consumers with transparent, trusted and secured data on goods and transactions and by contributing to a more equitable system of commerce for producers and consumers alike.

## 3 Edge-enabled MCS Platform for Data Collection and Rewarding in Pandemic Scenarios

This work focuses on an edge-enabled MCS platform targeted at supporting effective data collection/analysis and rewarding in critical scenarios, such as the recent COVID-19 one. In particular, we expanded our previous work described in [25] with two contributions: i) the design and development of an edge-enabled data collection/analysis module capable of evaluating the crowding degree of an area and ii) the development and testing of the edge-based distributed ledger for managing user's rewarding. Our edge-based extensions have been built and integrated within our MCS framework called ParticipAct [6].

ParticipAct is a comprehensive mobile crowdsensing platform developed the University of Bologna that provides us with the proper playground to gather crowd contributions and to test edge-based expansions in the crowdsensing domains of our interest. In ParticipAct the users have a sensing client application installed on their smartphones and they send collected information to a centralized cloud server, based on targeted sensing campaigns created by researchers and platform administrators. ParticipAct developers followed best practice guidelines in developing the crowdsensing platform. Thanks to the use of *MoST* [26], a high-performance sensing module, the ParticipAct client application has a very low footprint when running on devices, and it requires few actions from the user to collect data, avoiding boring him with requests. At any time, the user can stop the sensing data sharing and can reject tasks and campaigns that are proposed to him. The secure protection of users' data and the mechanisms for administrators and clients authentication guarantee the integrity of the profiles and contributions collected. Any user can freely view its own contributions to keep tabs on everything he is sharing with the community. The database replication assures the availability of data. The server side is built on top of open-source technologies, and its modularity permits easy expansion and reusability for different purposes in several domains, such as smart cities and transportation, people tracking, GPS data gathering, and trajectories drawing. Authorized administrators can create campaigns on the server and can choose the users to whom to propose them, the geo notification and geo activation areas, and a time frame during which the campaign is available. The many actions of a single campaign are called *Tasks* and they must all be completed before a user can declare a campaign concluded, send the collected data to the server, and possibly receive a reward for the quantity and quality of the information provided.

### 3.1 Data Collection and Crowding Degree Analysis

The basic cloud-based deployment of ParticipAct lacks some characteristics that are needed to address our requirement of providing a support for controlling the spread of a pandemia, such as in the recent COVID-19, by calculating the crowding degree of an area.

This requirement can be achieved with a massive data campaign supported by an effective data collection and user's rewarding management. The basic ParticipAct platform can involve many users around the country, but does not currently have the possibility to efficiently and effectively notify users in a specific geographic area with context-aware and location-aware updated information, being the cloud layer unaware of these data. The edge computing paradigm allows to overcome this limitation. Edge computing extends the cloud resources by offering networking, storing, computing capabilities and services distributed at the edge of the network closer to the final users. Edge nodes allow to reduce the load toward the server and the communication latency because they can perform local computation on data of interest and, most importantly, can promptly provide nearby users with location-aware information. Another limitation of the basic ParticipAct platform is the impossibility to federate different spontaneous systems spawned around the world. In this regard, we would like to achieve the purpose of sharing the user's scoreboard among ParticipAct federated servers deployed in distributed areas (ideally worldwide), maybe for different purposes. In this way, if a contributor should be involved in a crowdsensing campaign created by a server different from her usual one, she can contribute to the campaign and continue to acquire scores on the same profile, common among all federated servers. Thanks to the great customization feature of the platform, in [25] we started to formalize a way to federate different ParticipAct servers spawned around the world. Different servers, with possibly different purposes, can share the user rewards in order to enable the interaction of federated participants even if subscribed to different local servers. Users can benefit from the federation as they find their scores on any of the federated servers, regardless of the crowdsensing campaign they are participating in. Facilitating user's reward sharing among federated servers has a great beneficial impact on data collection. The catchment area participating in each campaign is increased making data collection more complete. In the case of a pandemia this is particularly important because it is possible to take into account the movement of users across different cities. When in a federated city users can still contribute to the campaign and user's presence can be still considered to precisely evaluate the crowding degree of an area.

Figure 1 shows the proposed edge-based architectural model of ParticipAct. In particular, each ParticipAct server relies on a pool of base MEC stations, as those defined by ETSI in the ETSI-MEC specification. We can assume that the exact position of the edge stations is always known by the server, and it is stored in a dedicated database containing the GPS coordinates of all associated MEC nodes. The participants in the crowdsensing campaigns have their own specific ParticipAct server to which they send contributes in order to complete the tasks assigned by ParticipAct's

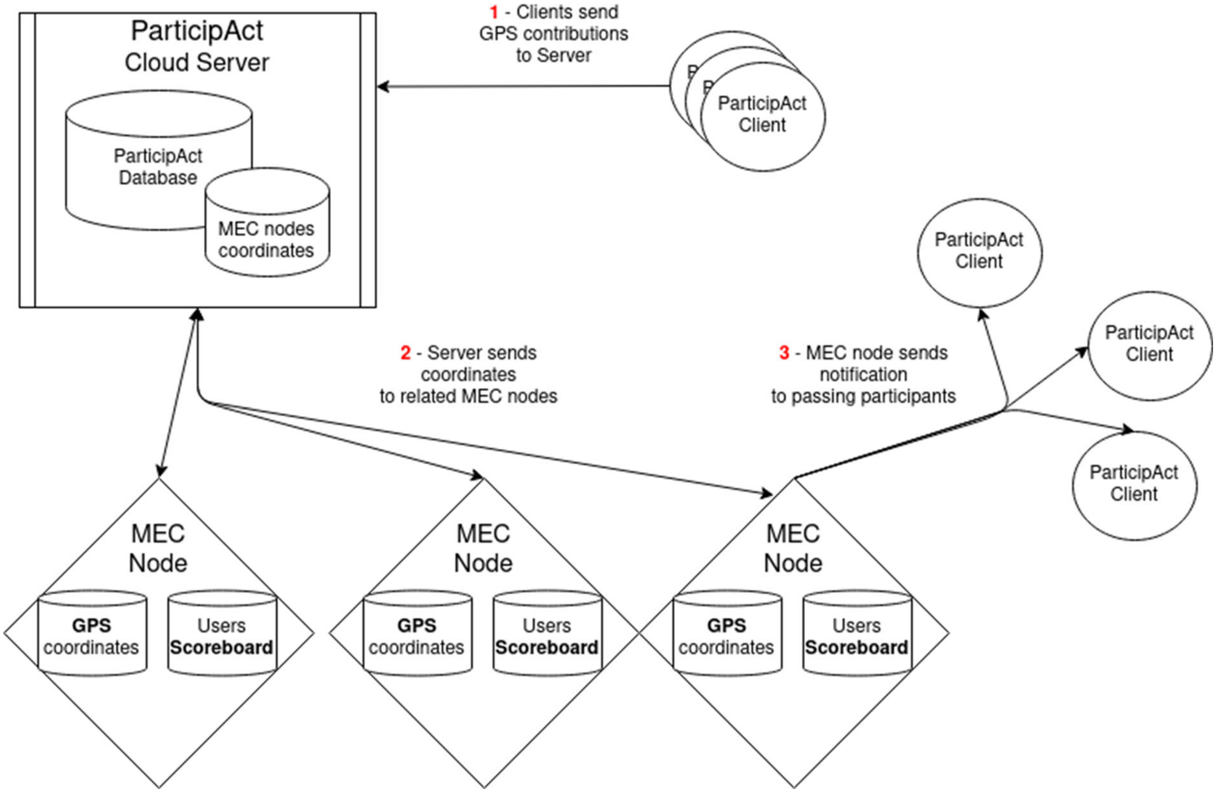

**Fig. 1** Edge-based participact architecture administrators and researchers. The ParticipAct platform already has all the tools to enable GPS tracking of users' locations with extreme precision. For the sake of controlling pandemic spreading, the administrators can choose the duration of a campaign. At campaign completion after participants can send all contributions. This policy is used to control the trade-off between monitoring level precision and network overhead. For example, a short campaign duration augments the precision of the contributions, on the contrary a long duration for a campaign decreases the network load.

For our use case, the contributors send to the server their GPS tracking location data created and stored on their devices while performing a GPS tracking task. In this way, the server keeps the contributions of all users in terms of GPS coordinates and areas visited by contributors during the tracking campaign.

As Section 4 will detail, from the ParticipAct's database containing all the contributions, it is possible to obtain information about most frequented areas in urban centers, outlining degrees of density, in terms of number of people, and classifying them based on the indications of the medical authorities. In our deployment, The ParticipAct server is hosted in a private datacenter or in a public cloud. However, during the crowdsensing campaigns, the server is engaged in a high number of tasks, including the receipt of contributions by users. Furthermore, a dataset of location contributions can reach the size of several tens of thousands of entries per day, and the calculation of the density could be a heavy computation task if performed at the server side. For these reasons, we decide to delegate the ParticipAct edge agents to perform the calculation of the high-density zones.

The ParticipAct server, based on an application-dependent policies, sends a subset of coordinates to each associated edge node for the density calculation. We recall that the server knows the positions of the MEC nodes, so it sends to a single edge node only the coordinates that pertain to the coverage area of the node. The policy with which this calculation takes place on MEC nodes depends on the policy according to which the server recursively sends contributions to the edge nodes. For example, if a server sends daily updates, the edge node will calculate the crowding of its related zones on a daily basis. At the end of a generic campaign, including those related to the pandemic control, in addition to the GPS coordinates, the ParticipAct servers will send to the associated edge nodes the prize to be awarded to each user who has completed a campaign, so that all MEC nodes will have a copy of user scores.

The MEC nodes now can notify people who pass within their range with alerts about crowded areas. This information will appear on all devices of people having ParticipAct application, but the data can be also shared with third-party health services to enrich the knowledge base of the smart city.

### 3.2 Edge-based Blockchain for Rewarding Management

In our proposal the storage of user's rewards in a federated crowdsensing environment is provided by an integrated edge-based blockchain platform within ParticipAct. The underlying reason for the adoption of a blockchain paradigm is to maintain rewards in a secure and distributed manner ensuring privacy and non-repudiable features. One crucial issue to consider when integrating a blockchain solution within a MCS platform is the architectural model to adopt. It is unrealistic to have a complete instance of the ledger on the end user devices and to rely on them for achieving a consistent ledger state. For saving resources we propose to rely on edge computing to distribute the ledger among multiple close-to-edge deployments. The ledger is distributed and decentralised among MEC nodes and MEC nodes are responsible for achieving a consistent state. We consider the employment of ETSI MEC nodes to improve the scalability of the entire system, in this way in fact the DLT-related functions can be executed exploiting the computing and storage edge resources lighten the cloud servers of in these additional tasks. In the edge-based blockchain architecture, shown in Fig. 2, the cloud server has a connection with a set of MEC nodes containing a full replica of the distributed ledger, i.e., all immutable concatenated rewards. The participant to the crowdsensing campaign is still afferent to an individual PartcipAct server to which a group of ETSI MEC nodes have been added. The MEC nodes have numerous services including a complete distributed ledger constituted by a full replica and a wallet service. The clients rely on the blockchain facilities provided by their closest MEC node and through it they can access and interact with the rewarding account records.

The federated infrastructure still remains for all intents and purposes unaltered, with collected data still privately kept by cloud servers independently from each other. After having validated a user's task result, cloud servers calculates and report its relative point allotment to their closest MEC nodes. This information is then added to the blockchain so that it can be then shared by every other MEC node under the control of federated members. More in details, when rewards need to be updated, MEC nodes execute a proper smart contract and validate the transaction containing the reward update request. In particular, MEC nodes perform the consensus protocol to add the new block containing the transaction to the blockchain and to maintain a consistent state of the ledger. In [25] we have compared the above described edge-based blockchain architecture with an alternative deployment solution based on the client-server model. In this case, as shown in Fig. 3, the distributed ledger is completely located at the server level, and it keeps the reward data in a distributed manner among federated nodes. The federation is constituted by all the organizations which take part of the the MCS campaign such as universities and company which constantly update the ledger in a way completely transparent to the end use. In this configuration the ledger can be placed aside of the ParticipAct database on each server keeping them independent and each reward update is broadcasted to every federated node.

Both the architectural approaches have different benefits and drawbacks. Comparing the client-server and an edge-based blockchain architectures as highlighted in the work [25], we can notice from the security perspective that the client-server architecture is potentially prone to tampering of the ledger since the number of federated institution servers tends to be low in the most common case. A low number of nodes enrolled in the server federation increases the risk of 50% + 1 attacks in which malicious actor can hijack the consensus protocol of the ledger taking over the majority of the nodes. Including the edge infrastructure in our ledger deployment improves the fault tolerance of the MCS platform, in this way in fact the blockchain knowledge base is distributed on many network segments which are more trustworthy

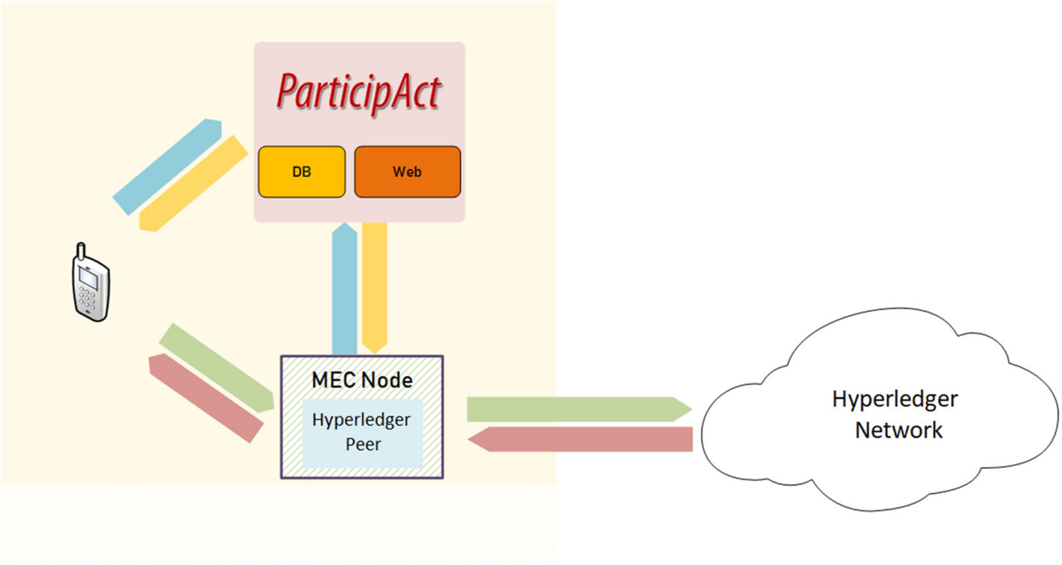

**Fig. 2** Edge-based blockchain architecture since are managed by third party's telecommunication providers.

## 4 Implementation

This Section provides a deeper view on the implementation and the algorithms executed in each single layer.

### 4.1 GPS Areas Density Calculation

We deployed our ParticipAct servers in the cloud layer. Each server relies on a pool of MEC base stations and has its own users. The clients always know the server's address because they registered with it. We used a private datacenter to run the server application, but being the server a classic web application, its installation can be made also in a public cloud.

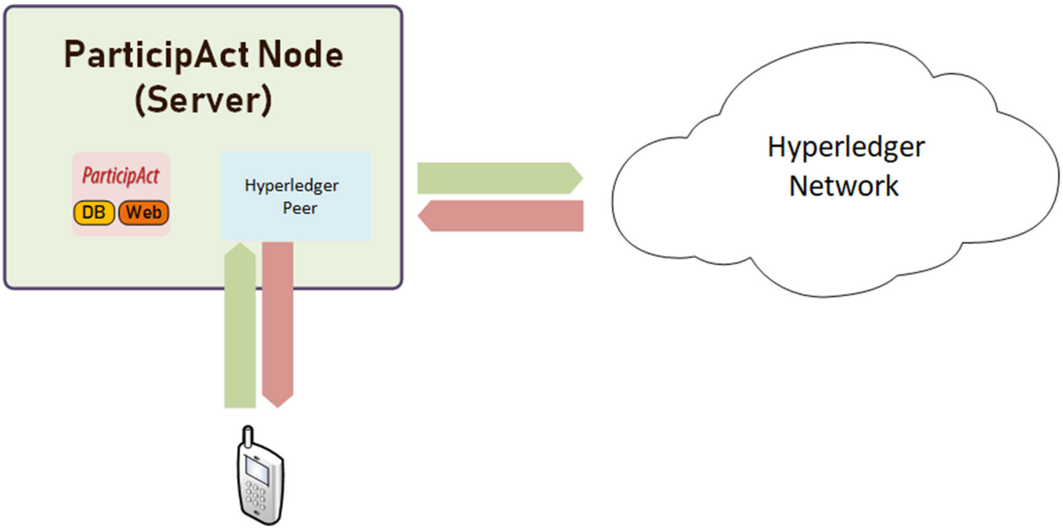

**Fig. 3** Client-server blockchain architecture

How we can see in Fig. 4, the ParticipAct server can create crowdsensing campaigns asking users to provide information from a broad range of sensors, including the GPS. By creating a GPS task, we can obtain information about the places people stay or pass-through.

The server is responsible for the storage, aggregation, and processing of GPS locations. From the locality data, we can infer the density of the areas visited by users of the platform. For the server, we chose the cloud deployment for the great availability of resources inside a datacenter. This choice gives us the scalability that high demanding operations need. However, the calculation of crowded areas will take place in each edge node associated with a server, for the reasons set out in Section 3. For the density area calculation, we started from the *datalocation* table stored inside the DB on which the ParticipAct server backend stores all user contributions. Table 1 is an excerpt of the *datalocation* table. We send part of the coordinates to each associated edge node, based on their coverage area.

We used the Geohash coordinates as an identifier of the area for which we want to classify the density of people in transit, we used the Geohash coordinates, a practical geocode system which uses a short string of digits and letters to encode a geographic area. Substantially this system breaks the earth surface into 32 sections, in turn, divided into other sub-regions identified with a unique string. The width of the area selected by a string depends on the size of the string, the longer the string, the smaller the selected area. This feature allows us to have a dynamic dimension of the areas in which we calculate the level of crowding. For the calculation of the Geohash from the ParticipAct *datalocation* table, we use the PostGIS extension (https://postgis.net/) for the PostgresSQL database. PostGIS enables geographic support to the database, allowing location queries to be run in SQL language.

```
ST_GeoHash(geometry geom, integer
maxchars=full_precision_of_point)
```

**Table 1** Excerpt from the *datalocation* table

| User_Id | Received timestamp | Latitude | Longitude |
|---|---|---|---|
| 24173 | 2017-01-01 00:02:17.384 | 37.4876 | 14.056 |
| 15697 | 2017-01-01 00:12:41.197 | 38.0989 | 13.3997 |
| 48360 | 2017-01-01 00:14:21.547 | 41.2776 | 15.2665 |
| 24173 | 2017-01-01 00:17:17.575 | 37.4922 | 14.0557 |
| 15697 | 2017-01-01 00:27:42.709 | 38.0989 | 13.3996 |
| 48360 | 2017-01-01 00:34:19.583 | 41.2776 | 15.2665 |
| 8659 | 2017-01-01 00:35:27.982 | 44.4974 | 11.3436 |
| 24173 | 2017-01-01 00:37:20.836 | 37.4925 | 14.056 |
| 15697 | 2017-01-01 00:47:41.573 | 38.0989 | 13.3997 |
| 24173 | 2017-01-01 00:52:16.757 | 37.4916 | 14.0528 |

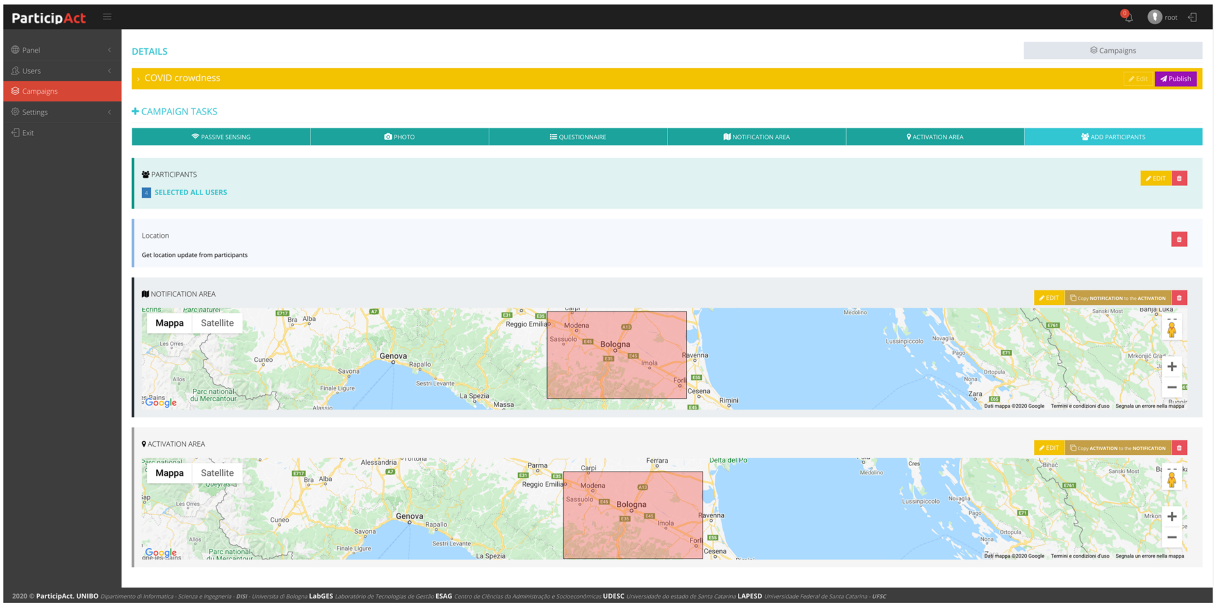

**Fig. 4** ParticipAct GPS geo notificated and geo activated campaign

The previous query realizes the transformation from coordinates to geo hashes, taking a *geometry* point and an integer for the precision. We recall that a shorter geo hash coincides with a larger zone (less precise). If no *maxchars* is provided, the algorithm uses the default maximum precision (20 characters). The first parameter, of geometric type, is created by the function **ST_MakePoint**.

```
ST_MakePoint(float long, float lat)
```

The function alone does not refer to any Spatial References Identifier (**SRID**), a unique unambiguous identifier associated with a specific coordinate system.

```
ST_SetSRID(ST_MakePoint(float long,
float lat),integer srid)
```

In our case, we use 4326 as SRID, which corresponds to the World Geodetic System 1984, used by GPS systems. The following query is the result of this algorithm using a geo hash area of 7 digits, or a tile size of 152.9 m x 152.4 m.

```
SELECT ST_GeoHash(ST_SetSRID(ST_MakePoi-
nt(long,lat),4326),7)
     INTO public.datalocationgeohash
     FROM public.datalocation;
```

We transformed all the coordinates in the *datalocation* table into geo hashes. We built a new table named *datalocationgeohash* taking the past coordinates gathered via GPS monitoring tasks. The last step to calculate the crowding of geo hash areas is the counting of the number of contributions for each area, our indicator of the population density in that area. The following query performs this operation.

```
SELECT st_geohash, COUNT (*)
   FROM public.datalocationgeohash
   GROUP BY st_geohash ORDER BY COUNT(*)
    DESC
```

## 4.2 Crowding Experimental Results

We investigate the capabilities of the ParticipAct server to calculate the crowding of geo hash areas, exploiting the ParticipAct dataset created through many crowdsensing real campaigns carried out from 2013 to 2017. We hypothesized that the density calculation takes place not continuously, but on a policy set on the basis of sending data from the server to the edge nodes. A fully functioning crowdsensing system, such as ParticipAct was during past data collection campaigns, could produce tens of thousands of entries containing user contributions. The geo hash calculation on so many entries could be a heavy process.

To obtain the following performance data we averaged ten runs of the SQL query to calculate the density. We simulated the two tiers, the server, and the edge one, using respectively a VM running in a private data center and having 4 vCPU, 16GB of RAM, and 100 GB of HD, and a laptop with quad-core Intel processor, 8 GB of RAM, and 20 GB of HD. We considered the contributions collected in the date 02/04/2014, so a table with 47384 GPS entries (coordinate points). We executed the algorithm showed in Section 3 on all the contributions of the selected day.

This calculation could not be negligible, indeed, usually, the server is under pressure during intensive crowdsensing campaigns due to the collection and processing of the data coming from the many sensors into the users' device. In this regard, we thought of transferring the processing of coordinates on the edge stations. Since each server relies on a pool of MEC Radio Access Network (RAN) stations, so the server sends part of the "datalocation" table (latitude, longitude, and contributions) to each edge site, based on their position. For example, if a RAN station covers a certain geographic area, only the positions involved in its range will be sent to that station. This cuts down on the calculation times of crowded areas as each edge station would only have to do with the contributions of users who have passed through this location. For this experiment, we used geo hashes with 7 digits, covering an area of 150 square meters, and we hypothesized a RAN coverage area of about 300 meters.

Table 2 shows the overhead due to the calculation of the densities performed respectively on the server and on the edge. The edge had to process only coordinates that belong to its coverage area, which are only 3754 entries. Instead, the server has to process all the contributions of the day. Although we simulated the MEC stations with a less powerful asset, they show a

**Table 2** Performance comparison between cloud and edge deployments during geo hashes and density calculation

| Deployment | Time (sec) | Rows number | CPU usage | RAM usage |
|---|---|---|---|---|
| Cloud | 5.42 | 47384 | 97% | 0.5% |
| Edge | 0.511 | 3754 | 90% | 0.4% |

lighter footprint on system resources with respect to the execution on the server, due to the limited number of contributions on which to act. The timing for the edge case does not take into account the data splitting between the edge nodes, because it is carried out on the cloud side once a day and with negligible timing compared to the total gain.

Table 3 is the result of the calculation of the crowding of geo hash areas under the coverage of a hypothetical RAN MEC station located at the engineering faculty of the University of Bologna. The first column represents the geo hash areas under the edge station coverage, whereas the second column reports the number of people passing through that area during the analyzed day.

### 4.3 Reward Implementation

For the choice of the most suitable blockchain platform, we have evaluated the main distributed ledger (DL) and their features such as permissioned vs permissionless, tokenized vs tokenless below. Permissioness blockchain means that users need prior approval (credentials like certificates or keys) before take part of the ledger and using it submitting transactions and smart contract whereas a permissionless blockchain lets anyone participate in the system. The second analyzed feature is about tokenized DL which has a mechanism to generate the currency like mining and require fee for transactions. Tokenized DL allow to exchange the currency for fiat currencies and requires a lot of computing power. The tokenless DL does not have any fee or mining mechanism and are prone to spam. The blockchain platforms analyzed for our use case are: Hyperledger Fabric and Ethereum. Fabric is a permissioned tokenless blockchain framework originally contributed by IBM and hosted by the Linux Foundation. Ethereum is a tokenized distributed ledger which provides digital money, data services and distributed applications, it supports permissionless and permissioned deployments and the use of a self-executing code known as smart contracts run by Ethereum Virtual Machine (EVM).

For our solution the Hyperledger Fabric platform turned out to be best suited to our needs thanks to its permissioned and tokenless feature which better support the private nature of our rewards network.

The Hyperledger Fabric ledger is constituted by two different parts: world state and blockchain. The world state is a database which keeps the current values of the attributes of an object represented by key-value pairs. The world state allows the programs a quick access to the blockchain values without having to go through the entire blockchain to calculate it. The second is the blockchain transaction log which keeps the transaction history collected in blocks. Each block contains a cryptographic hash of the previous block, a timestamp, and transaction data generally represented as a Merkle tree. In Hyperledger Fabric, each node have a copy of the world state and the blockchain ledger and any update of the ledger is performed by the peers individually executing the consensus algorithm. This algorithm is at the base of the consistency and ensures that every update is executed in a uniform manner on each peer and all the peers have identical copies of the ledger.

Fabric defines three types of peers which are all involved in the consensus protocol with different: i) endorsed peers which receive and validate the transactions and execute smart contracts; ii) ordered peers which create transaction blocks and receive endorsed transaction proposals and insert them in a block together with others in an orderly manner; iii) committer peers which receive and broadcast transactions and blocks. In addition, Hyperledger Fabric offers the chaincode (the equivalent of the Ethereum smart contract) implementation in different general-purpose programming languages which are Java, Go, and Node.js. This feature makes easy the development of smart contract because we don't need to learn another language avoiding an additional layer of abstraction at programming level.

The application that manages the users' rewards is based on a Hyperledger Fabric chaincode and is written in Go which is the most performing language as highlighted in the work [27]. It maintains the rewards as a matrix data structure of triple

**Table 3** Geo hash area's density calculation on a single edge node

| Geo hash area | Crowding |
|---|---|
| srbj1v8 | 657 |
| srbj1eh | 537 |
| srbj1g9 | 477 |
| srbj1dz | 376 |
| srbj1tr | 329 |

<identifier:integer:timestamp>; the identifier is unique and is used by the ParticipAct application to anonymize the user personal data, the integer represents the number of points per user while the timestamp keeps track of the most recent data upload constituting a simple invalidation mechanism on blockchain. The reward chaincode is accessed in writing only from the server that calculate the increment or decrement of the gamification points based on task user contribution and it is accessed in reading from any participant user to check the score. A reward update on the ledger starts always by the server which evaluate the client gamification task and attributes a score to the user sending a transaction proposal to the MEC nodes. The MEC nodes reply with the responses which are received by the server and sent to the orderer node. The orderer first determinates the satisfaction of the endorsement policy and then compare the answers. The endorsement policy defines the number of peers which have to execute the chaincode to validate the transaction. The reading operation on the ledger to get the reward score can be rather executed by all the clients sending a transaction proposal query and immediately getting the result.

### 4.4 Hyperledger Fabric Chaincode Experimental Results

Since the chaincode performance is crucial for a responsive and secure reward mechanism, we have analyzed and evaluated the transaction latency at varying of the number of participating endorser peers. The endorser peers play a crucial role in the consensus algorithm because are the nodes on which the chaincode is installed, they receive and execute the transaction proposal sent from the clients and reply sending back endorsed result (endorsed transaction proposal).

We defined the transaction latency as the time between when the client sends the request and when the response is received. Analyzing the Fabric operation, we can calculate two different type of latency: the query latency and the update latency which depend on the type of operation executed. For a query in fact the interaction is only between client and a peer, while an update involves also endorser, orderer and committer peer and goes through all the consensus algorithm phases. For these reasons we differentiate between *Query Latency (LpQ)* which is the time interval waited by the client between the sending of the request and the receiving of the response and the *Update Latency (LpT)* which is the time interval waited by the client between the request sending and the receiving a confirmation event to notice the inclusion of the transaction in a block and then to the blockchain.

Both *LpQ* and *LpT* are composed of a sum among several latencies, for the *LpQ* they are: (1) the time taken by the client application, (2) the time taken by a transaction to reach the number of endorser peers declared in the endorsement policy and to get the response, (3) the overall chaincode execution time, (4) the number of concurrent transactions executed, (5) the time spent to read/write the worldstate. For the *LpT* other two latencies are added: (6) the time taken by the orderer to receive transactions and organize them in blocks, (7) the amount of time to receive the new block, validate all the transaction individually and include it to each ledger peer. The latency is influenced by many factors such as, for example, peers and client machine hardware, network latency. Other important factors are the number of endorser peers and endorsement policy which defines the number of endorser peers that have to execute the transaction. Moreover, there is the type of orderer service that can work in one mode or Kafka, in the first one only a single node is involved while Kafka requires coordination between the different orderer nodes. Crucial is also the chaincode implementation details and programming language.

The testing scenario is constituted by VMs connected by a local network and created on a OpenStack-based cloud infrastructure constituted by 4 server hosts. Each VM has 2 CPUs, 2 GB RAM and 20GB of disk and runs Ubuntu 16.04 LTS and Hyperledger Fabric (version 1.4). All the endorser peers run on different VMs, they have the chaincode installed and use LevelDB as world state. The orderer is implemented in one mode and runs on another distinct VM, while the CA is in a separate container. The client runs on a further VM which belongs to the local network. The tests are designed to measure the query and the update latency at varying of the number of peers. The tests uses the Go languages both for the client and for the chaincode at varying the number of nodes of the network. The number of nodes for the tests are 1, 2, 4, 8, 10, 12, 14 and 16 nodes and consider the worst case

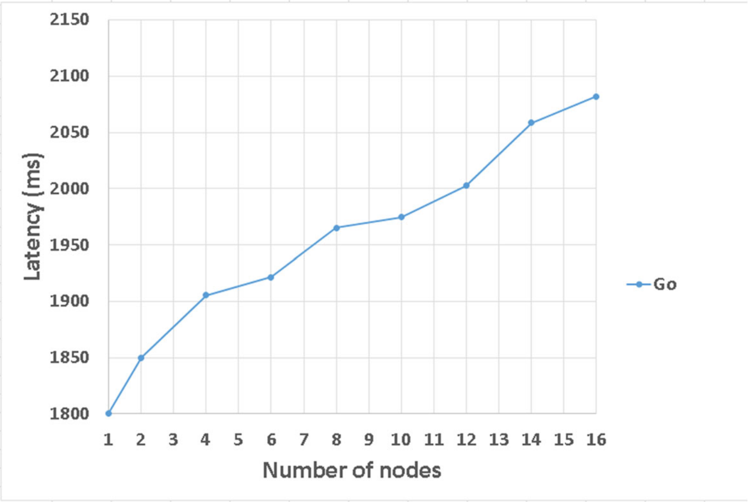

**Fig. 5** Update transaction latency which is when the transaction proposal is requested and executed by all the network peers to satisfy the endorsement policy.

The evaluation was carried out on the ledger both for the *Update Latency (LpQ)* and for *Query Latency (LpQ)*. For each experiment the client performs 50 transactions in sequence, the interval measured calculates the time from the request sending request to the response receiving. We have repeated each experiment 33 times to reduce error factor and in the graphs are shown the average values; we have not reported the standard deviations which are always below 6% for all tests. We consider the reception of the result for the experiment related to the queries while for the experiments focused on the updates the notification related to the inclusion of a transaction to a block and the appending on the blockchain is considered. To perform a write, we executed a writing of a new reward score for a certain user while for reading we requested the reward points of the same user.

Figure 5 shows the latency related to a ledger update, the transactions modify the worldstate and are then included to the blockchain. The update is then propagated to all the peers. We can notice that the graph follows a linear trend, it starts from about 1800 ms and grows up to 2100 ms. The difference of 300 ms is caused by the execution of consensus algorithm and, in particular, the transaction proposal which have to be executed on all the 16 nodes to satisfy the endorsement policy.

Figure 6 shows the latency related to the queries which exploits only the first phases of the Hyperledger Fabric consensus algorithm without modify the ledger including the transactions. Differently from above in

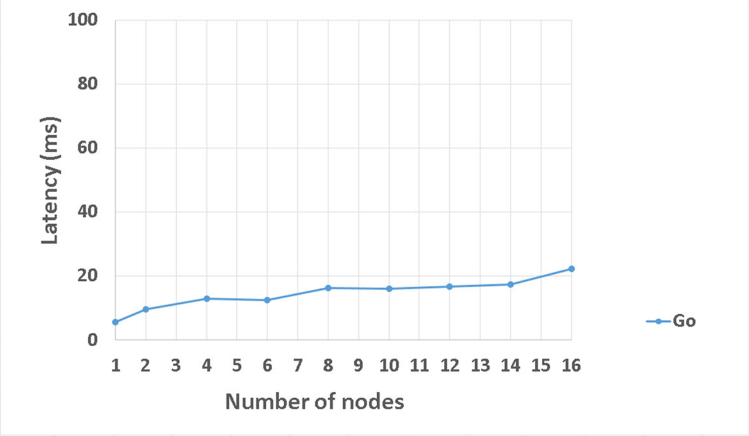

**Fig. 6** Query transaction latency fact the reply does not have to wait for the replies of all the network nodes interacting only with a single peer. As we can see from the graph, in this case, the trend at the varying of the number of the peers is approximately constant, specifically, the latency starts from about 5 ms and grows up to 20 ms.

Comparing the graphs, we can easily notice that the latency for a query is noticeably lower than for an update of the ledger. In particular, its average time is about 100 times faster than an update transaction execution. As described before, we believe the reason can be researched in the nature of the consensus algorithm and in the way the data is included into the blockchain. In the case of update in fact the algorithm involves several communications and an agreement among peers, in the second case the information is directly generated by a unique peer.

## 5 Conclusions

Mobile crowdsensing is a paradigm that empowers the citizen contributions melting this data with smart city information. Pervasivness of mobile and wearables devices gives good chances to obtain detailed data from the crowd. Modern smart cities need for scalable and interoperable solutions in order to achieve a precise model of the reality and to give citizen and public institutions a good support based on real data. The gamification among the participants to crowdsensing campaigns improves the quality and the quantity of contributions provided by platform users.

We have extended the classic client-server infrastructure of MCS platforms by adding the edge layer and we have proposed a blockchain mechanism to federate different MCS servers in order to increase the catchment area and participation in crowdsensing campaigns. The edge layer supports both a local processing of location data on MEC nodes associated with each server and a full-distributed permissioned blockchain platform to keep the reward user score. Thanks to the edge we have numerous advantages. On the one hand it allows us to free the server from complex calculations that involve a non-negligible overhead. On the other hand, it increases the robustness of the distributed ledger platform replicating it on many nodes. In case of potential server failure the users' scores could be recovered from any replica on an associated edge node.

We tested these new capabilities using our MCS platform, namely ParticipAct, extending it with the middle edge tier and proving its operability. We specialized the platform by adding the capability to find crowded areas based on the presence of users in those areas during the crowdsensing campaigns. This integrationcan be used to mitigate the spread of pandemic diseases and could be of help, both for the smart city and for citizens, during the current health emergency concerning the spread of COVID-19. The calculation of the most crowded areas is a complex operation that involves many transformations from geographical coordinates to geo hashes values identifying a zone with different precision. In our tests, we put the MEC nodes under stress with the calculation of crowded areas, based on users' positions, and with the updating of the blockchain to estimate the users' score.

We are planning to completely integrate the edge capabilities with our crowdsensing platform model. The next step could be the implementation of our logic inside a real MEC node, following the official ETSI specifications. We will implement the activity recognition to programmatically and dynamically adjust the precision of the crowded radius areas calculated by our algorithm, with the hope that the users contributions could help in improving smart interactive services in the healthcare field and in situations of great emergency such as the one we are experiencing today.

**Funding** Open access funding provided by Alma Mater Studiorum - Università di Bologna within the CRUI-CARE Agreement.

**Open Access** This article is licensed under a Creative Commons Attribution 4.0 International License, which permits use, sharing, adaptation, distribution and reproduction in any medium or format, as long as you give appropriate credit to the original author(s) and the source, provide a link to the Creative Commons licence, and indicate if changes were made. The images or other third party material in this article are included in the article's Creative Commons licence, unless indicated otherwise in a credit line to the material. If material is not included in the article's Creative Commons licence and your intended use is not permitted by statutory regulation or exceeds the permitted use, you will need to obtain permission directly from the copyright holder. To view a copy of this licence, visit http://creativecommons.org/licenses/by/4.0/.
